# Enhanced Pb(II) Adsorption by a Ternary MnO_2_/NH_2_-MIL-101(Fe)/Graphitic Carbon Nitride Nanocomposite Through Complementary Interfacial Interactions

**DOI:** 10.3390/polym18182277

**Published:** 2026-09-17

**Authors:** Faten M. Ali Zainy, Amr A. Yakout

**Affiliations:** Department of Chemistry, College of Science, University of Jeddah, P.O. Box 80237, Jeddah 21589, Saudi Arabia

**Keywords:** Pb(II), metal–organic framework (MOF), graphitic carbon nitride, adsorption, nanocomposite

## Abstract

Severe heavy metal pollution in aquatic environments demands the engineered development of structurally optimized, highly selective adsorbents. Herein, we report the intentional fabrication of a novel ternary MnO_2_/NH_2_-MIL-101(Fe)/*g*-C_3_N_4_ nanocomposite via an integrated in situ interfacial growth pathway. The true architectural novelty of this multi-component assembly lies in utilizing the 3D mesoporous metal–organic framework (MOF) scaffolding to structurally isolate the 2D *g*-C_3_N_4_ layers and prevent the self-aggregation of redox-active MnO_2_ nanoparticles. Comprehensive characterization via PXRD, FTIR, TEM, Raman, and high-resolution XPS confirmed that this unique interfacial hybridization maximizes the spatial exposure of unblocked chemical binding sites. Batch extraction trials demonstrated a high Pb^2+^ removal efficiency of 99.5 ± 2.7% at an optimized pH of 6.0, yielding a superior maximum monolayer adsorption capacity (*q*_max_) of 431.8 mg.g^−1^. Competitive selectivity matrices containing co-existing ions (Cu^2+^, Cd^2+^, Ni^2+^, and Cr^3+^) revealed noticeable selectivity toward Pb^2+^ ions, driven by soft Lewis’s acid-base affinities, while background electrolyte tests identified SO_4_^2−^ as the most influential competing anion. Non-linear isotherm modeling showed a better agreement with the Langmuir model, suggesting dominant monolayer adsorption on accessible surface sites, while kinetic data followed the pseudo-second-order model. Spectroscopic profiling proved that this heightened performance is dictated by a multi-modal integrated network operating via cooperative inner-sphere Mn-OH complexation, oxygen-vacancy trapping, exocyclic framework amine (-NH_2_) chelation, and *g*-C_3_N_4_ triazine dative configurations. These findings establish the ternary system as an advanced, highly recyclable benchmark for targeted heavy metal decontamination.

## 1. Introduction

Accelerated global industrial growth alongside intensifying urbanization continues to drive the release of anthropogenic toxic heavy metals directly into aquatic systems [1]. These discharges present severe, systemic hazards to human health, native biological systems, and broad ecosystem integrity [1]. Regulatory bodies like the U.S. Environmental Protection Agency (EPA) closely monitor and enforce stringent permissible caps on these elements due to their acute environmental persistence [2,3,4,5]. Among these regulated contaminants, divalent lead (Pb^2+^) requires effective remediation strategies [2,3,4,5]. It is completely non-biodegradable, chemically stable, and prone to rapid bioaccumulation across trophic webs [2,3,4,5]. The primary entry vectors for aquatic Pb^2+^ pollution involve unmitigated industrial wastewater discharges, mining runoff, improper electronic waste disposal, and failing municipal sewage treatment infrastructure [1]. Chronic exposure to trace Pb^2+^ concentrations results in permanent physiological damage in humans [6]. This includes severe renal failure, developmental abnormalities, and neurological impairments [7]. Consequently, finding practical, high-efficiency remediation setups to isolate trace Pb^2+^ from complex aquatic environments remains a vital objective for modern environmental engineering [6,8].

Wastewater decontamination typically depends on several standard treatment routes. These include chemical precipitation, membrane separation, ion exchange, and photocatalytic degradation [9,10,11,12,13]. Among these options, adsorption processes are widely preferred for scaling up trace decontamination [14]. This preference stems from their low operating costs, simplicity, high processing efficiencies, and minimal secondary byproduct output [14]. Standard sorbents, including activated carbons, natural clays, polymers, chitosan, and layered double hydroxides (LDHs), have seen extensive use [15,16,17,18,19,20]. Despite this, their real-world deployment is frequently constrained by poor thermodynamic selectivity, low structural durability, and a sharp drop in capture efficiency when handling trace contaminant levels [15,16,17,18,19,20]. Furthermore, certain organic matrices face chemical degradation when exposed to aggressive wastewater conditions, introducing risks of secondary contamination [21]. These limitations emphasize the need to develop advanced, structurally resilient materials featuring tailored surface chemistries designed for target-specific heavy metal immobilization.

Metal–organic frameworks (MOFs) represent a powerful class of crystalline porous materials characterized by long-range order, huge specific surface areas, adjustable pore configurations, and highly customizable surface environments [22,23]. For example, functionalized networks like citric anhydride-modified NH_2_-MIL-53(Al) achieve a Pb^2+^ uptake capacity of 90.8 mg.g^−1^ via distinct monolayer chemisorption pathways [24]. Similarly, oxygen-functionalized 3D architectures facilitate rapid Pb^2+^ capture driven by strong coordination and electrostatic attraction [25]. Beyond these examples, diverse MOF platforms (including Cd, Tb, Cu, and Zr-based variations) [26,27,28,29,30,31,32] and their derivatives are widely utilized to capture various ecological threats, ranging from bisphenols and polychlorinated biphenyls to organophosphate esters [33,34,35] and toxic mercury species [36]. Within this family, iron-centered NH_2_-MIL-101(Fe) serves as an exceptionally water-stable, low-toxicity, and robust bifunctional framework. The dense distribution of active surface amine (-NH_2_) groups within its accessible mesoporous grid offers ideal coordination sites for binding target metal ions. This is demonstrated by its effectiveness in extracting arsenate/arsenite ions [37] and its use in magnetic silica composites designed for simultaneous Pb^2+^ and Cd^2+^ immobilization [38]. Furthermore, the specific optical characteristics of NH_2_-MIL-101(Fe) allow its application as a fluorescent sensing platform capable of identifying Fe^3+^, Cu^2+^, and Pb^2+^ ions at remarkably low detection limits [39,40,41].

Nevertheless, moving from idealized pristine MOF crystals to practical water purification frameworks introduces significant engineering bottlenecks. A primary limitation is the common tendency of isolated MOF particles to undergo severe self-aggregation in water, which buries active coordination sites and compromises mass transfer kinetics. Moreover, many existing studies focus predominantly on analytical-scale sensing or qualitative monitoring rather than high-capacity bulk decontamination under dynamic hydrodynamic settings [42]. To circumvent these problems, hybridizing secondary functional nanostructures within a parent MOF matrix provides a viable path forward. Graphitic carbon nitride (*g*-C_3_N_4_), a two-dimensional conjugated polymer rich in basic nitrogen groups, exhibits excellent chemical, thermal, and mechanical durability [42,43,44,45,46]. However, pristine *g*-C_3_N_4_ suffers from a restricted surface area because its individual layers pack tightly together [47,48]. Intercalating or combining *g*-C_3_N_4_ with specialized nanoparticles helps prevent this layer stacking, which opens the internal porosity and markedly enhances overall extraction yields [49,50,51,52]. Simultaneously, nanoscale manganese dioxide (MnO_2_) is widely appreciated as a highly economical, bio-friendly sorbent phase. It displays a strong thermodynamic affinity for heavy metals through mechanisms of surface complexation, ion exchange, and localized chemical precipitation, operating optimally within the mildly acidic pH ranges common to industrial waste streams [53,54,55,56,57,58].

Building on the complementary properties of MnO_2_, NH_2_-MIL-101(Fe), and *g*-C_3_N_4_, this work reports the synthesis and adsorption performance of a ternary MnO_2_/NH_2_-MIL-101(Fe)/*g*-C_3_N_4_ nanocomposite for Pb(II) removal from aqueous media. The design rationale is based on combining oxygen-rich MnO_2_ sites, the nitrogen-containing framework of g-C_3_N_4_, and the amine-functionalized porous structure of NH_2_-MIL-101(Fe) within a single hybrid adsorbent. Unlike binary MnO_2_/MOF systems, where partial aggregation of MnO_2_ may reduce the exposure of oxide active sites, or g-C_3_N_4_/MOF composites, where restacking of carbon nitride layers can limit accessibility, the ternary structure provides a more integrated interfacial environment. Similarly, MnO_2_/*g*-C_3_N_4_ hybrids offer both oxygen- and nitrogen-based binding groups but lack the ordered three-dimensional MOF scaffold required to maintain dispersion and support rapid ion transport. In the present material, NH_2_-MIL-101(Fe) acts as a porous host that helps distribute MnO_2_ and g-C_3_N_4_ while preserving accessible diffusion pathways. This arrangement exposes multiple Pb(II)-binding domains, including Mn–OH/Mn–O groups, Fe–O clusters, exocyclic –NH_2_ groups, and triazine-type nitrogen sites, which may contribute cooperatively to metal-ion uptake through complexation, coordination, electrostatic attraction, and pore-assisted diffusion.

The adsorption behavior of the nanocomposite was evaluated under different operational conditions, including solution pH, adsorbent dosage, contact time, competing metal ions, and background electrolytes. Kinetic and equilibrium models were also applied to clarify the dominant uptake behavior and adsorption capacity. Through this combined experimental and modeling approach, the study provides insight into how the ternary architecture improves site accessibility, reduces phase aggregation, and promotes Pb(II) immobilization. Overall, the work presents a rational material-design strategy for developing MOF-based hybrid adsorbents with enhanced selectivity, reusability, and performance for heavy metal removal from water.

## 2. Experimental Section

### 2.1. Chemicals and Solutions

All chemical precursors and analytical-grade reagents were utilized immediately upon receipt without undergoing additional purification steps. Sinopharm Chemical Reagent Co., Ltd. (Shanghai, China) supplied the precursor bulk graphitic carbon nitride (*g*-C_3_N_4_) powder along with the organic ligand 2-aminoterephthalic acid. Organic solvents, including methanol (CH_3_OH), ethanol (C_2_H_5_OH), glacial acetic acid (CH_3_COOH), and N,N-dimethylformamide (DMF) were obtained from Honeywell. Sigma-Aldrich (St. Louis, MO, USA) provided the regulatory pH adjustment solutions, hydrochloric acid (HCl), 36 wt% and sodium hydroxide (NaOH). The same vendor supplied the high-purity metal salts needed to prepare stock solutions and evaluate competitive ion effects: lead nitrate [Pb(NO_3_)_2_], copper(II) nitrate trihydrate [Cu(NO_3_)_2_.3H_2_O], chromium(III) nitrate nonahydrate [Cr(NO_3_)_3_.9H_2_O], nickel(II) nitrate hexahydrate [Ni(NO_3_)_2_.6H_2_O], cadmium nitrate [Cd(NO_3_)_2_], and iron(III) chloride hexahydrate [FeCl_3_.6H_2_O]. Background electrolyte salts and potential interferences, including sodium chloride (NaCl), sodium sulfate (Na_2_SO_4_), sodium dihydrogen phosphate (NaH_2_PO_4_), and potassium permanganate (KMnO_4_), were also sourced from Sigma-Aldrich. Ultrapure water (18.2 MΩ.cm), generated via a Milli-Q filtration apparatus, served exclusively as the solvent medium throughout all aqueous reactions, solution preparations, and post-synthetic washing procedures.

### 2.2. Instrumentation

Quantification of residual Pb^2+^ and alternative co-existing heavy metal ions was primarily conducted via flame atomic absorption spectrometry (FAAS) utilizing a PerkinElmer PinAAcle 900F spectrophotometer (PerkinElmer, Shelton, CT, USA). For experimental trials yielding trace or ultra-low metallic concentrations exceeding standard FAAS detection bounds, samples were analyzed via inductively coupled plasma mass spectrometry (ICP-MS). Textural properties of the synthesized materials, specifically specific surface area, average pore diameter, and total pore volume, were evaluated through nitrogen (N_2_) adsorption–desorption isotherms at 77K on a Quantachrome Brunauer–Emmett–Teller (BET) analyzer (Boynton Beach, FL, USA). Morphological features and surface topography were examined using a JEOL JSM-6010LV (JEOL, Tokyo, Japan) scanning electron microscope (SEM). Surface functional group profiles were mapped via Fourier transform infrared spectroscopy (FTIR) on a Nicolet 400 spectrometer (Nicolet, Madison, WI, USA) across a spectral wavenumber range of 4000 to 400 cm^−1^. Crystalline phase distribution, structural integrity, and bulk phase purity were verified by powder X-ray diffraction (XRD) recorded on a Rigaku D/MAX-2550 (Rigaku, Tokyo, Japan) diffractometer. To determine near-surface elemental states, coordination environments, and chemical compositions, X-ray photoelectron spectroscopy (XPS) was conducted with a Thermo ESCALAB 250Xi spectrometer (Thermo Fisher Scientific, Waltham, MA, USA) at an instrumental energy resolution of 0.5 eV. Supplemental structural features, carbon hybridizations, and electronic configurations were recorded via Raman spectroscopy using a Thermo Scientific™ DXR3 Flex Raman Spectrometer (Thermo Fisher Scientific, Madison, WI, USA).

### 2.3. Preparation of MnO_2_/NH_2_-MIL-101(Fe)/g-C_3_N_4_ Nanocomposite

The multi-component MnO_2_/NH_2_-MIL-101(Fe)/*g*-C_3_N_4_ system was engineered via a dual-stage process. First, the crystalline NH_2_-MIL-101(Fe) framework was isolated using solvothermal crystallization [59]. Second, nanoscale manganese dioxide (MnO_2_) was loaded onto the coordinated NH_2_-MIL-101(Fe)/*g*-C_3_N_4_ matrix through in situ reduction. To form the initial framework [59], 2.70 g of iron(III) chloride hexahydrate (FeCl_3_.6H_2_O) was completely dissolved in a mixed solution of deionized water and N,N-dimethylformamide (DMF) via mechanical stirring. In a separate beaker, 1.00 g of the structural ligand 2-aminoterephthalic acid was fully dispersed in ethanol using 20-min of sonication. This organic linker dispersion was then slowly added to the iron precursor liquid. To guarantee bulk homogeneity, the combined system was stirred continuously for 45 min. The resulting solution was then sealed inside a 100 mL Teflon-lined stainless-steel autoclave. The solvothermal reaction ran at 150 °C for exactly 12 h. Following cooling to room temperature, the solid product was isolated via centrifugation. This solid was rinsed with DMF, ethanol, and deionized water until all remaining impurities were removed. The wet powder was dried under vacuum at 80 °C for 12 h to finalize the activated NH_2_-MIL-101(Fe). Separately, bulk graphitic carbon nitride (*g*-C_3_N_4_) was produced by pyrolyzing melamine powder inside a furnace at 520–550 °C for 3–4 h. To integrate these two distinct phases, the prepared NH_2_-MIL-101(Fe) and *g*-C_3_N_4_ powders were combined inside a stainless-steel ball-milling reactor and processed at a vibration frequency of 30 Hz for 20 min. The final deposition of the MnO_2_ nanoparticles was achieved via directed in situ chemical precipitation [60,61]. The newly generated MnO_2_/NH_2_-MIL-101(Fe)/*g*-C_3_N_4_ powder was suspended in alcohol and deoxygenated by bubbling high-purity nitrogen (N_2_) gas through the liquid for 10 min. A burette was then used to introduce a 0.1 M potassium permanganate (KMnO_4_) solution into the vortex in a slow, dropwise manner while maintaining rapid stirring. This process triggered the localized conversion and precipitation of MnO_2_ nanoparticles directly within the open matrix of the MnO_2_/NH_2_-MIL-101(Fe)/*g*-C_3_N_4_ substrate [60,61]. This reduction sequence was allowed to proceed under ambient temperature conditions for 30 min, ensuring an ultra-fine, uniform distribution of the oxide nanoparticles across the structural scaffolding. Ultimately, the completed ternary MnO_2_/NH_2_-MIL-101(Fe)/*g*-C_3_N_4_ nanocomposite was separated by centrifugation, washed thoroughly with alternating cycles of ethanol and water, and dried completely under vacuum at 60 °C.

### 2.4. Pb(II) Batch Adsorption Study, Regeneration and Application Tests

Isothermal batch extraction trials were performed by dispersing exactly 50 mg of the ternary MnO_2_/NH_2_-MIL-101(Fe)/*g*-C_3_N_4_ matrix into 100 mL aliquots of Pb^2+^-spiked aqueous solutions. To ensure adequate phase boundary contact and reach equilibrium, the sealed vials were agitated at 250 rpm inside a thermostatic shaking incubator held at a fixed temperature of 25 °C for 40 min. Following the termination of the contact interval, the spent solid sorbent was isolated from the liquid phase via centrifugation. Initial screening of residual concentrations was tracked using turbidity metrics, while definitive concentrations of targeted metal ions, specifically Cu^2+^, Cd^2+^, Ni^2+^, Cr^3+^, and Pb^2+^, were measured via flame atomic absorption spectrometry (FAAS) or inductively coupled plasma mass spectrometry (ICP-MS). Multi-point calibration protocols for Pb^2+^ yielded highly linear curves (*R*^2^ > 0.995). All experiments were executed in triplicate, and data points reported herein represent calculated mean values derived from independent runs. Systematic evaluation of the Pb^2+^ immobilization process was conducted using an initial metallic concentration of 25 mg L^−1^ to observe the independent influences of phase acidity, sorbent dose, and competing ionic species. For the boundary pH optimization profiles, the aqueous phase was altered between pH 2.0 and 10.0 through the dropwise addition of 0.1 *M* HCl or NaOH solutions. Background electrolyte and co-ion interference effects were determined by incorporating representative sodium salts of common inorganic anions, including chloride (Cl^−^), dihydrogen phosphate (H_2_PO_4_^−^), and sulfate (SO_4_^2−^), with each background species introduced at a concentration range of 0–0.2 m*M*. Competitive selectivity matrix tests containing an equimolar blend of Cu^2+^, Cd^2+^, Ni^2+^, Cr^3+^, and Pb^2+^ ions were carried out at optimized benchmark parameters: an operating pH of 6.0, an active composite mass of 50 mg, and an equilibration duration of 40 min. Sorbent stability and reusability profiles were established across consecutive, identical adsorption–desorption operations. Following each specific extraction phase, the spent solid was separated and dried under vacuum for 12 h. Stripping of the bound heavy metals was achieved by immersing the isolated powder into 20 mL of a combined 0.1 *M*-HCl/0.05 *M*-EDTA eluent solution, followed by agitation at 250 rpm for 5.0 min at 25 °C. The regenerated ternary nanocomposite was then collected, washed with ultrapure water, dried completely, and reintroduced into subsequent target capture runs. Comparative tracking of the pollutant removal percentages before and after stripping allowed for the quantification of material structural integrity and long-term recycling performance. The representative percentage removal efficiency (%*R*) and the equilibrium capture capacity (*q*_e_, (mg.g^−1^) were determined using the standard mass-balance relationships outlined in Equations (1) and (2), respectively:

(1) $$\%\;R=\;\;\frac{C_{o}-\;C_{t}\;}{c_{o}}\;\;\times \;\;100$$

(2) $$\%\;q_{t}=\;\frac{{(C}_{o}-C_{t})\;V\;}{m}$$

where *C*_0_ and *C*_t_ (mg L^−1^) represent the initial target metal ion concentration and the concentration remaining at time *t*, respectively, *V* (L) is the solution volume, and *m* (*g*) is the mass of the nanocomposite used in the adsorption experiment. All adsorption measurements were carried out in triplicate, and the reported values correspond to the average of the independent experiments. The analytical performance of the method was assessed in terms of precision and accuracy through replicate determinations using recovery values and relative standard deviation (RSD) as evaluation criteria.

## 3. Results and Discussion

### 3.1. Morphological, Microstructural and Elemental Composition of MnO_2_/NH_2_-MIL-101(Fe)/g-C_3_N_4_

**Figure 1:**
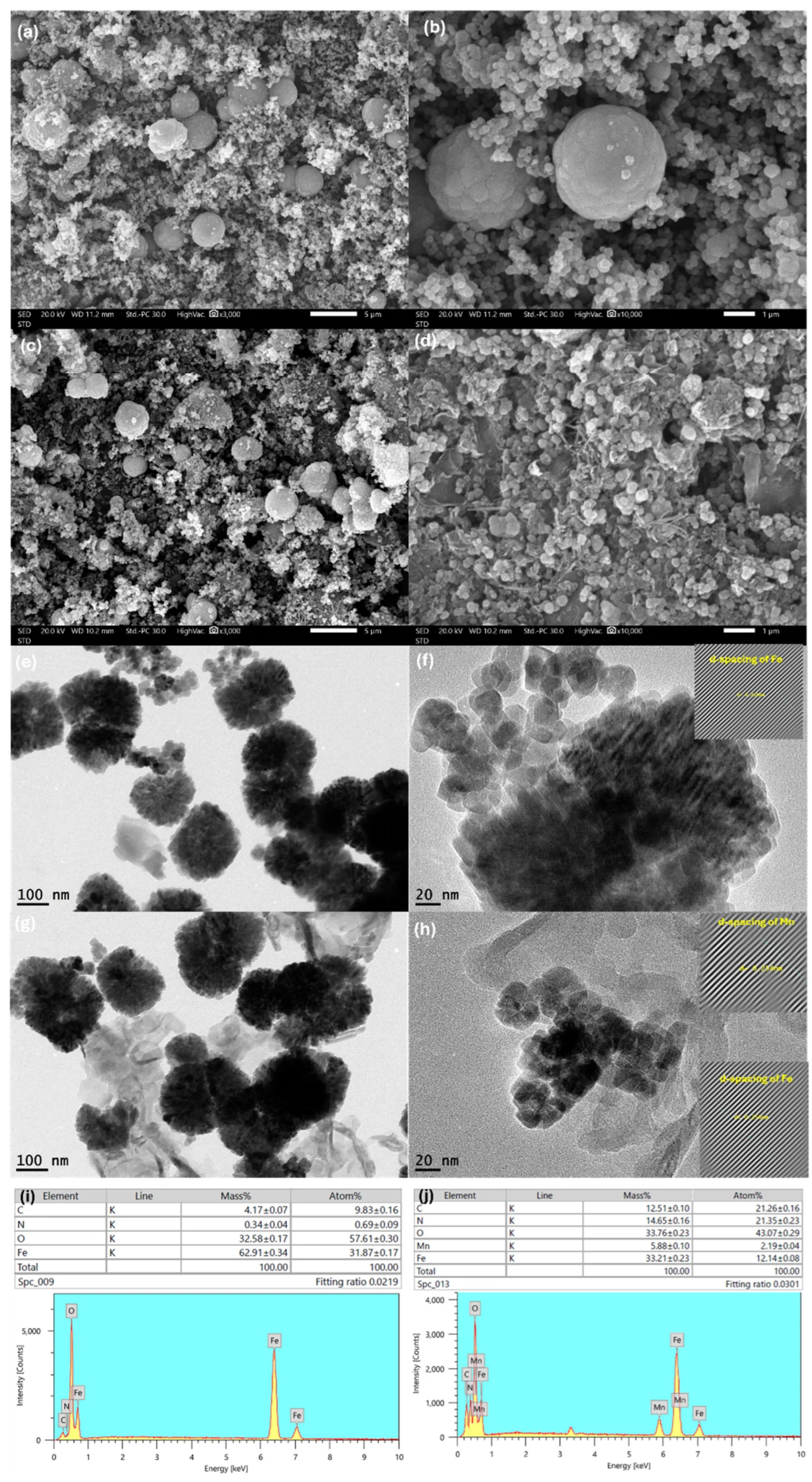
SEM (**a**,**b**) and (**c**,**d**), TEM (**e**,**f**) and (**g**,**h**), EDX (**i**,**j**) of NH_2_-MIL-101(Fe) and MnO_2_/NH_2_-MIL-101(Fe)/*g*-C_3_N_4_ nanocomposites, respectively.

The surface architecture and microstructural evolution of the isolated building blocks and the final ternary MnO_2_/NH_2_-MIL-101(Fe)/*g*-C_3_N_4_ framework were systematically examined using electron microscopy. SEM scans of the pristine NH_2_-MIL-101(Fe) framework (Figure 1a,b) reveal highly defined, well-crystallized octahedral micro-crystals with smooth facets and clean boundaries. This morphology confirms the successful assembly of the secondary building units under the optimized solvothermal conditions. In contrast, the SEM micrographs of the fully integrated ternary nanocomposite (Figure 1c,d) showcase a noticeable morphological transition. The original smooth facets of the octahedral MOF crystals are heavily populated by a continuous, highly wrinkled layer of nanoscale sheets and interconnected particulate clusters. This structural change indicates that the layered *g*-C_3_N_4_ sheets and the in situ precipitated MnO_2_ nanoparticles form a secure coating across the MOF, creating a rougher, highly accessible multi-component interface.

To investigate the internal phase boundaries and confirm crystallization at the atomic scale, TEM characterizations were conducted. The narrow-focus TEM views of the core NH_2_-MIL-101(Fe) framework (Figure 1e,f) confirm the clean octahedral solid geometries observed in the SEM images, showing high structural transparency without any noticeable pore blockages. For the finished ternary nanocomposite (Figure 1g,h), the TEM images reveal that the nanoscale MnO_2_ fractions and thin *g*-C_3_N_4_ polymer sheets are securely distributed over the underlying MOF surfaces. An essential structural fingerprint is resolved within the distinct lattice fringes identified via HRTEM modeling of the hybrid interface:

(i) The core framework areas reveal well-resolved, long-range lattice spacings measuring exactly 0.86 nm, which correspond perfectly to the primary, open (022) reflection planes of crystalline NH_2_-MIL-101(Fe). This structural persistence demonstrates that the internal framework channels remain intact and uncollapsed after the subsequent synthesis steps.
(ii) Directly across the hybridized exterior zones, distinct, narrower lattice fringes with a d-spacing of 0.24 nm emerge. This specific atomic spacing matches the characteristic (101) crystal planes of nanoscale MnO_2_.

The direct alignment of these two distinct lattice planes at the interface confirms that the nanoscale oxides are chemically anchored to the MOF support rather than existing as a simple physical blend. This deliberate structural configuration helps prevent the natural self-aggregation of MnO_2_ and suppresses the stacking of 2D carbon nitride layers. As a result, the structural integrity of the composite is preserved, maintaining a highly stable and open pore distribution that facilitates rapid target ion diffusion during decontamination.

The elemental composition of the prepared materials was investigated by energy-dispersive X-ray spectroscopy (EDX) to confirm the presence of the expected elements and evaluate the successful incorporation of the different components. The EDX spectrum of the pristine NH_2_-MIL-101(Fe) framework (Figure 1i) displays characteristic signals corresponding to Fe, C, N, and O, confirming the elemental composition of the synthesized MOF and the absence of detectable foreign elements. After the multi-step synthesis, the EDX spectrum of the ternary MnO_2_/NH_2_-MIL-101(Fe)/g-C_3_N_4_ nanocomposite (Figure 1j) shows the characteristic signals of Fe, C, N, and O, along with an additional Mn peak. The appearance of the Mn signal confirms the successful incorporation of the MnO_2_ phase into the composite structure. These results, together with the XRD and spectroscopic analyses, support the successful formation of the intended ternary nanocomposite.

The MnO_2_/NH_2_-MIL-101(Fe)/*g*-C_3_N_4_ nanocomposite exhibited a high BET surface area of 872.31 m^2^ g^−1^, with an average pore width of 21.55 nm and a total pore volume of 0.5215 cm^3^ g^−1^. The monolayer adsorption volume, expressed as the amount of gas adsorbed at STP, was estimated to be 23.71 cm^3^ g^−1^, confirming the presence of a highly accessible porous surface suitable for efficient adsorbate uptake.

**Figure 2:**
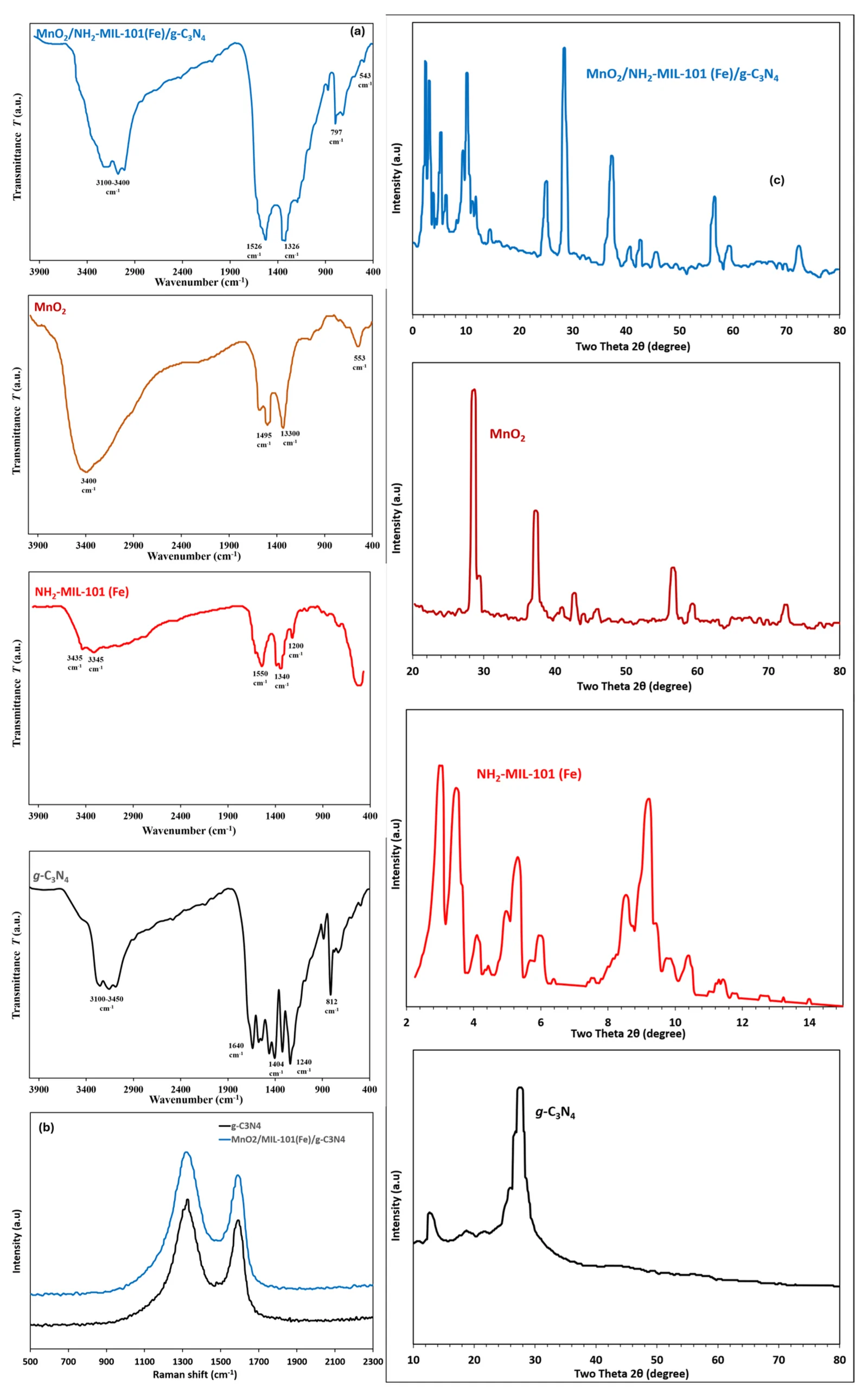
FTIR (**a**), Raman spectra (**b**), XRD (**c**) of MnO_2_/NH_2_-MIL-101(Fe)/*g*-C_3_N_4_ nanocomposite.

The FTIR spectrum (Figure 2a) confirms the successful integration of NH_2_-MIL-101(Fe), g-C_3_N_4_, and MnO_2_ by displaying the characteristic vibrational features of the individual components. The NH_2_-MIL-101(Fe) framework exhibits two characteristic absorption bands at 3435 and 3345 cm^−1^, which are assigned to the asymmetric and symmetric stretching vibrations of the primary exocyclic –NH_2_ groups, respectively [59]. The bands observed at 1550 and 1340 cm^−1^ are attributed to the asymmetric and symmetric stretching vibrations of the coordinated carboxylate groups (COO^−^), confirming the coordination between the organic linker and Fe-based metal clusters [59]. The successful incorporation of the *g*-C_3_N_4_ phase is evidenced by the characteristic absorption bands located in the 1200–1640 cm^−1^ region, corresponding to the stretching vibrations of aromatic C–N and C=N heterocycles, while the band at 812 cm^−1^ is assigned to the breathing vibration of the triazine/heptazine ring structure [60]. Furthermore, the low-frequency absorption band at 543 cm^−1^ is attributed to the characteristic metal–oxygen vibrations of Mn–O and Fe–O bonds, confirming the incorporation of the MnO_2_ phase within the composite structure [61,62]. The coexistence of these characteristic vibrational bands demonstrates the successful formation of the ternary MnO_2_/NH_2_-MIL-101(Fe)/g-C_3_N_4_ nanocomposite.

Complementary surface structural fingerprints were resolved using Raman profiling (Figure 2b). The graphitic carbon nitride component yields distinctive shifts at 1315 cm^−1^ and 1565 cm^−1^, which align with the classic disordered (D-band) and crystalline carbon (G-band) vibrations of sp^2^-hybridized graphitic sheets. The underlying MOF network is confirmed by sharp Raman modes at 1435 cm^−1^ and 1610 cm^−1^, which originate from the localized ring stretching of the organic aminobenzoate linkers [63]. Additionally, a distinct vibrational peak appears at 642 cm^−1^. This signature is assigned to the symmetric stretching vibration of Mn-O bonds within the MnO_6_ octahedral coordination blocks, providing clear evidence that the crystalline manganese oxide phase is well-dispersed across the composite surface.

The physical long-range crystallinity and structural composition of the product were evaluated via powder XRD tracking (Figure 2c). The recorded pattern preserves the sharp, narrow reflections below 2θ = 15°, which match the standard simulated crystallographic data for the NH_2_-MIL-101(Fe) cubic phase [60,64]. This retention proves that the hosting MOF maintains its high structural stability and porous network during the subsequent processing steps. The incorporation of the organic sheet phase is indicated by a broad reflection centered at 2θ = 27.4°, which corresponds to the characteristic (002) interplanar stacking distance of conjugated graphitic carbon nitride layers. Moreover, the successful crystal nucleation of the metal oxide is validated by distinct peaks appearing at 2θ = 37.1° and 66.2°, which correspond to the (101) and (111) reflection planes of crystalline MnO_2_ [65]. The smooth co-existence of these three sets of diffraction peaks confirms that the ternary hybrid material was successfully synthesized with all components securely integrated at the nanoscale.

**Figure 3:**
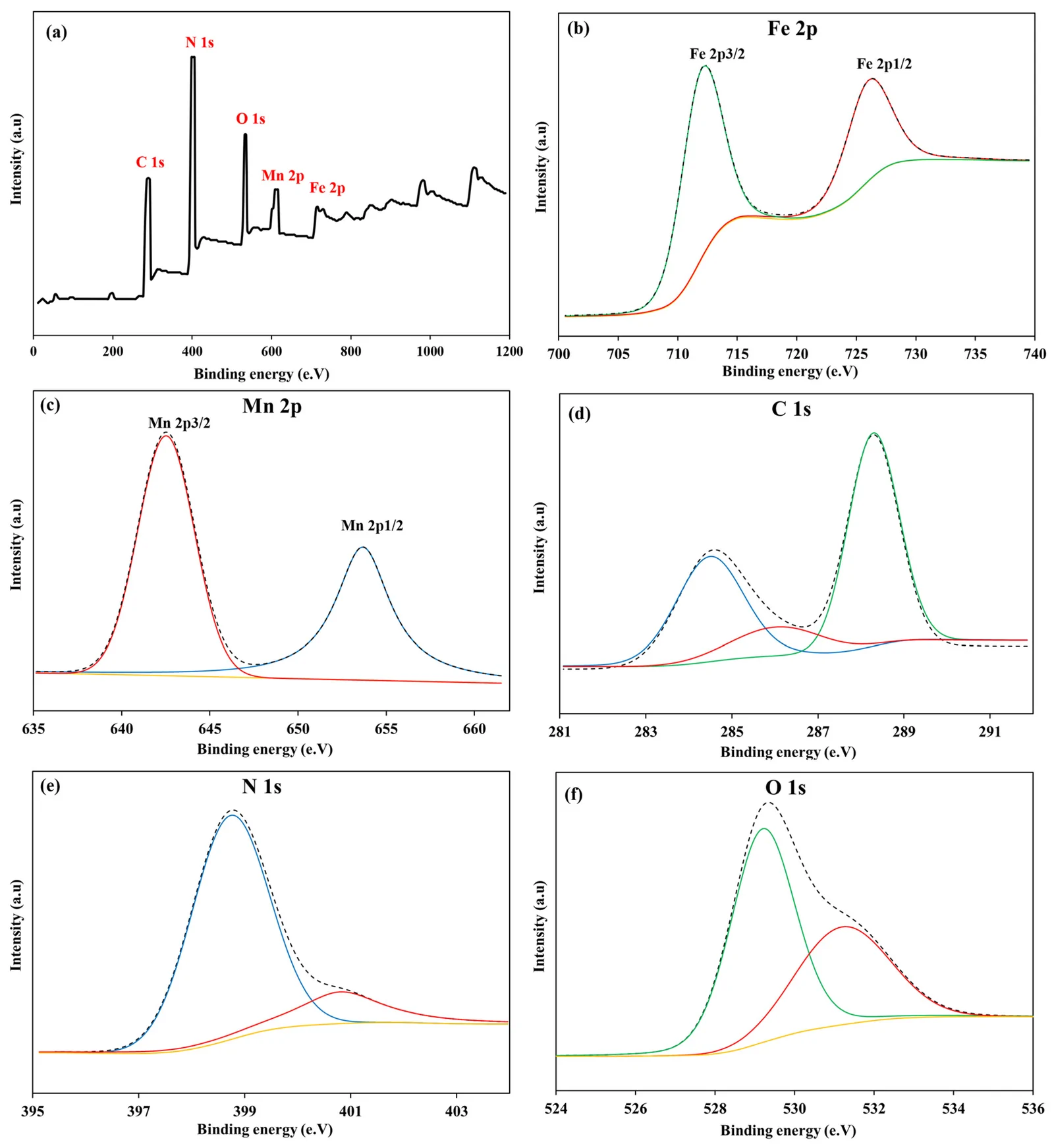
Full spectrum XPS (**a**), high-resolution XPS spectra of Fe 2p, Mn 2p, C1s, N1s, and O 1s (**b**–**f**) of MnO_2_/NH_2_-MIL-101(Fe)/*g*-C_3_N_4_ nanocomposite.

Surface composition and interfacial binding mechanisms within the ternary MnO_2_/NH_2_-MIL-101(Fe)/*g*-C_3_N_4_ nanocomposite were elucidated through comparative pre- and post-adsorption XPS analyses. Wide-scan survey profiling (Figure 3a) confirmed successful ternary hybridization via characteristic emissions for C 1s, N 1s, O 1s, Fe 2p, and Mn 2p. Narrow-scan C 1s spectra (Figure 3d) resolved distinct carbon environments corresponding to adventitious C-C/C=C (284.8 eV), amine-bound C-N (286.4 eV), and framework triazine/carboxylate N-C=N/O-C=O components (288.5 eV), all of which preserved structural positions following metal capture to show framework stability [66,67]. In contrast, the high-resolution N 1s core levels (Figure 3e) revealed notable positive binding energy shifts (+0.2 to 0.3 eV) in both the pyridinic C-N=C (398.7 eV) and exocyclic primary amine -NH_2_ (401.2 eV) states, indicating substantial electron transfer from nitrogen lone pairs to vacant orbitals on incoming Pb^2+^ ions through deliberate coordination. This chelation pathway is supported by the O 1s deconvolution (Figure 3f), where the surface metal-bound hydroxyl M-OH) signal at 531.4 eV shifted upward and experienced a sharp drop in relative peak area, demonstrating that inner-sphere oxygen-metal complexation and localized ion-exchange happen simultaneously during decontamination [68]. Furthermore, oxidation state fingerprints remained constant for the structural nodes, as evidenced by the fixed Mn 2p doublet (Figure 3c, Mn 2p3/2 at 642.1 eV; Δ*E* = 11.7 eV), validating a stable Mn^4+^ valence in the MnO_2_ phase, and the Fe 2p profile (Figure 3b, Fe 2p3/2 at 711.6 eV) with its shake-up satellite confirming a preserved Fe^3+^ coordination state [69]. Together, these spectroscopic shifts demonstrate that lead immobilization on the hybrid material is dictated by a multi-modal chemisorption network, operating via cooperative interfacial coordination across the active amine, triazine nitrogen, and oxide-linked hydroxyl sites.

### 3.2. Batch Adsorption Study

#### 3.2.1. Impact of pH

**Figure 4:**
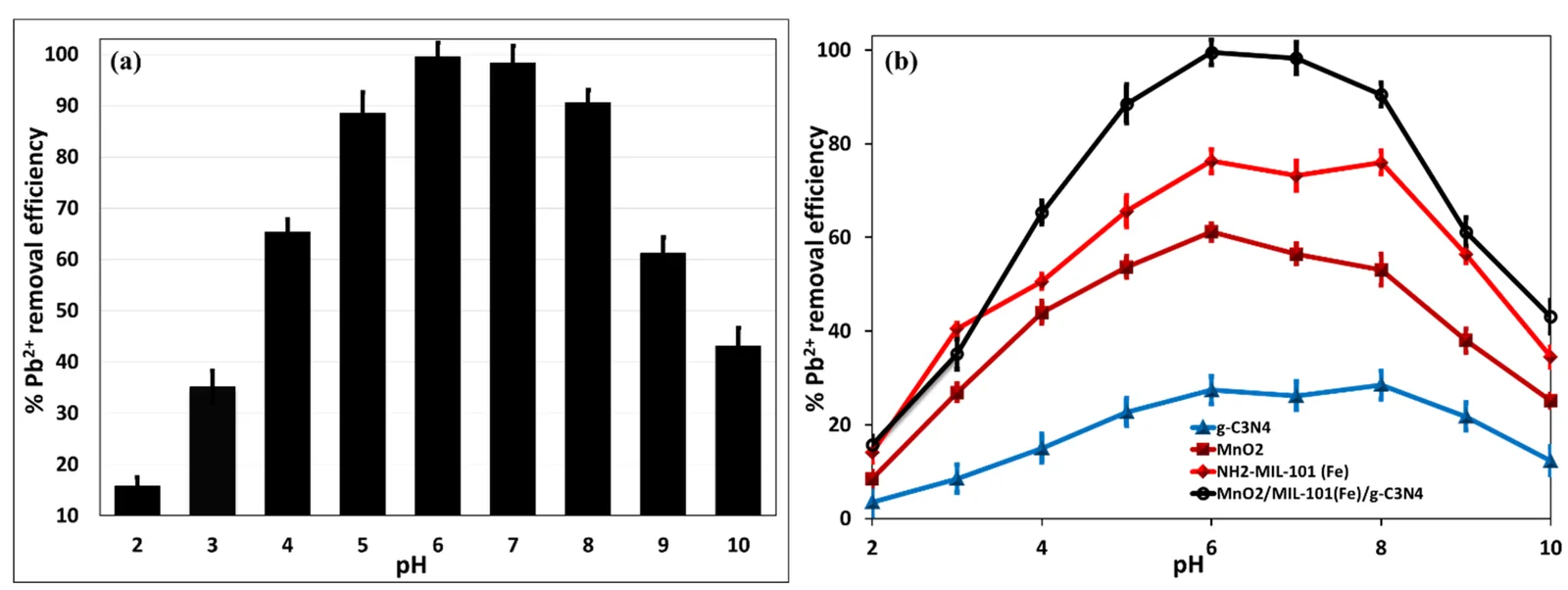
The effect of pH on the Pb^2+^ removal efficiency by the MnO_2_/NH_2_-MIL-101(Fe)/g-C_3_N_4_ nanocomposite (**a**), with a comparative study for the impact of each moiety (**b**) in the MnO_2_/NH_2_-MIL-101(Fe)/g-C_3_N_4_ nanocomposite on the Pb^2+^ removal efficiency at different pH values, and at the same optimum conditions.

The dependency of Pb^2+^ removal efficiency on solution pH explicitly reflects structural modifications in surface charge, active site availability, and metal speciation across the ternary MnO_2_/NH_2_-MIL-101(Fe)/*g*-C_3_N_4_ framework (Figure 4a). In highly acidic media (pH < 4.0), dominant hydronium ions protonate the active exocyclic amine (-NH_2_) and triazine (-C=N) networks, yielding a highly positive surface charge that induces severe electrostatic repulsion against incoming, free hydrated Pb^2+^ cations. As the system shifts from mildly acidic to neutral conditions (pH 4.0–7.5), structural deprotonation occurs alongside a sharp decline in competing H_3_O^+^ ions, creating an optimal operational plateau where the maximum Pb^2+^ capture efficiency peaks precisely at pH 6.0, yielding a high removal value of 99.5 ± 2.7%. Comparative profiling reveals that this outstanding performance stems from interconnected multi-component interactions; Figure 4b. Within this network, the MnO_2_ fraction drives inner-sphere complexation, ion-exchange, and interlayer trapping via its dense surface hydroxyls (Mn-OH); the open NH_2_-MIL-101(Fe) scaffolding provides ultrahigh porous channels for rapid mass transport while exposing open Fe-O cluster nodes and electron-rich amine centers; and the *g*-C_3_N_4_ sheets supply aromatic pyridinic nitrogen lone pairs to establish stable N→Pb^2+^ dative binding sites alongside supplemental cation-π configurations. Beyond pH 7.5, observed increases in removal metrics are no longer dictated by pure surface adsorption but rather by macroscopic hydrolysis, causing physical particle agglomeration and the chemical precipitation of insoluble lead hydroxides.

#### 3.2.2. The Impact of Constituent Mass Ratio of the Nanocomposite on the Pb^2+^ Uptake Performance

**Figure 5:**
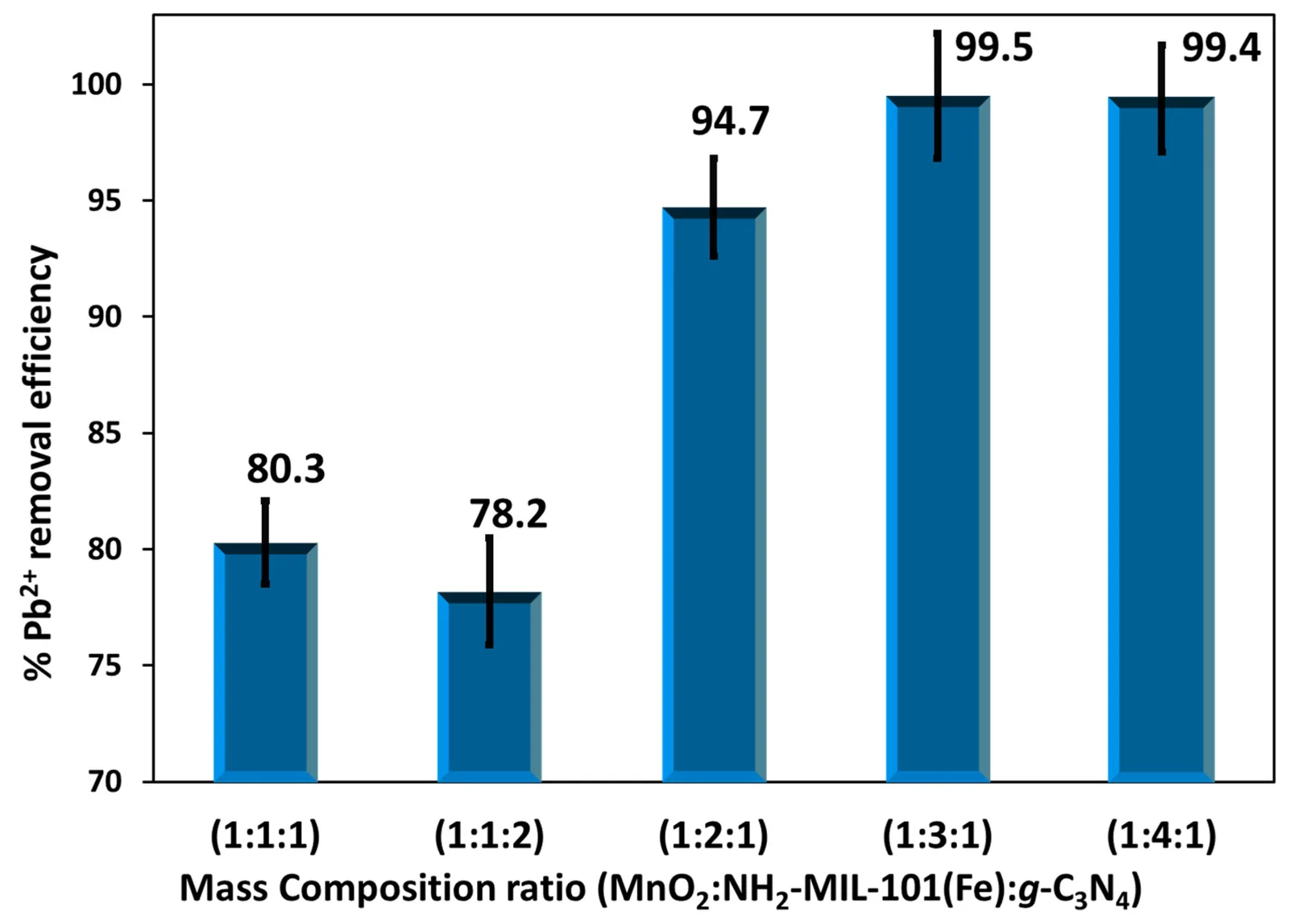
The impact of constituent mass ratio of the nanocomposite on the % Pb^2+^ uptake performance.

The optimization of the ternary MnO_2_/NH_2_-MIL-101(Fe)/*g*-C_3_N_4_ nanocomposite was systematically pursued by preparing five distinct mass configurations, 1:1:1, 1:2:1, 1:3:1, 1:4:1, and 1:1:2, to investigate how varying the structural presence of each component governs the overall Pb^2+^ uptake (Figure 5). Batch extraction trials identified the 1:3:1 mass ratio as the definitive peak composition, delivering a superior lead removal efficiency of 99.5 ± 2.7%. From a structural standpoint, this formulation establishes an ideal interfacial equilibrium between the exposed active sites and the available internal pore volume. Increasing the iron-based NH_2_-MIL-101(Fe) content up to a factor of three (moving from 1:1:1 to 1:3:1) noticeably escalates pollutant immobilization; this trend occurs because the open, 3D mesoporous architecture of the MOF functions as a robust structural skeleton that prevents the raw stacking of 2D *g*-C_3_N_4_ sheets and the self-aggregation of MnO_2_ nanoparticles. By keeping these active phases highly dispersed, the 1:3:1 framework maximizes the exposure of localized chemical binding sites, specifically the electron-rich primary amine groups NH_2_), triazine nitrogen rings (-C=N), and highly reactive oxide-linked surface hydroxyl centers (Mn-OH). Conversely, pushing the MOF content past this threshold to a 1:4:1 ratio, or doubling the carbon nitride content to 1:1:2, causes a noticeable drop in performance; this decline is attributed to mass-transport blockages where an excess of one component fills the open channels, causing localized aggregation that buries coordination centers and restricts fluid diffusion. Because it balanced structural accessibility with high site density, the optimized 1:3:1 composition was chosen as the baseline material for all subsequent solid-state characterizations, kinetic studies, and isothermal modeling.

#### 3.2.3. Impact of the Nanocomposite Mass Dosage

**Figure 6:**
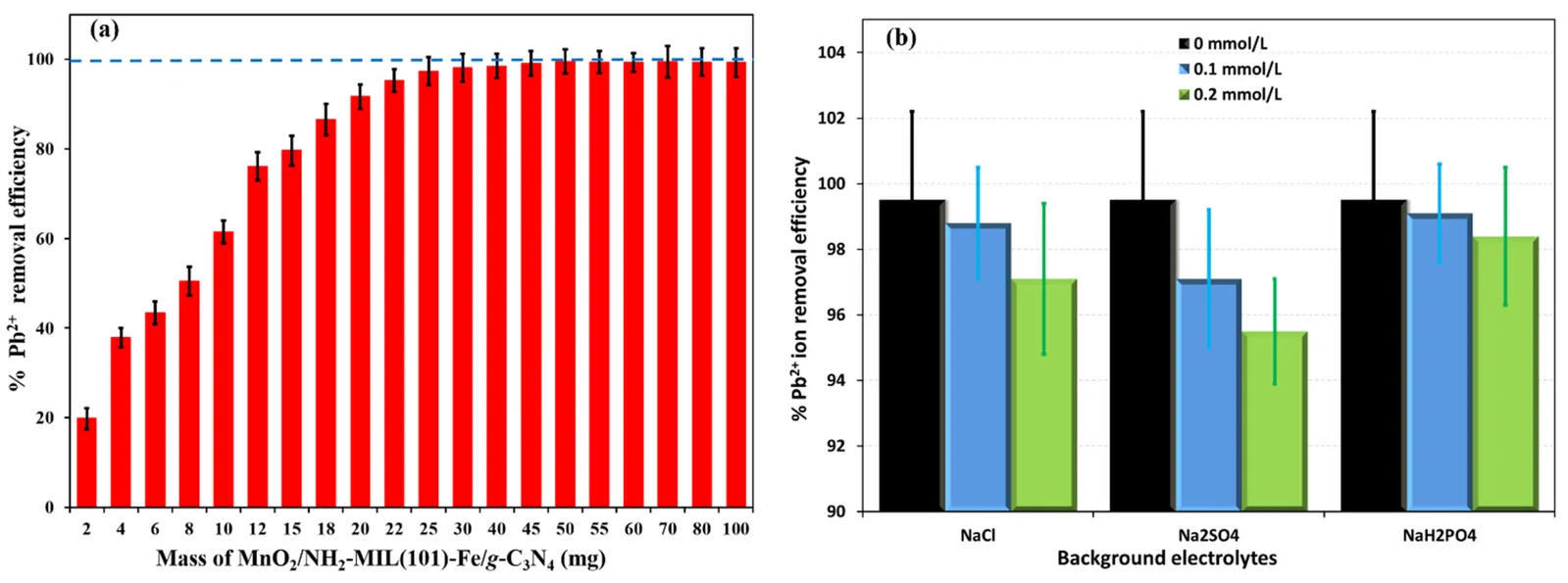
Effect of nanocomposite mass (mg), (**a**) and ionic types and concentration, (**b**) on the % Pb^2+^ removal efficiency.

The effect of MnO_2_/NH_2_-MIL-101(Fe)/*g*-C_3_N_4_ nanocomposite mass on Pb(II) removal was examined at pH 6 over the range of 1–100 mg, as shown in Figure 6a. The removal efficiency increased markedly with increasing adsorbent mass, rising from 19.8% at the lowest dosage to approximately 99.5% at higher dosages. This trend is mainly attributed to the progressive increase in the number of available adsorption sites and the larger accessible surface area provided by the nanocomposite. At low adsorbent mass, the available binding sites are insufficient to capture all Pb(II) ions present in solution, resulting in limited removal. As the nanocomposite mass increases, more active sites become accessible, enhancing Pb(II) uptake through surface binding interactions. However, after 50 mg, no considerable improvement in removal efficiency was observed, and the adsorption response reached an almost steady plateau. This indicates that most Pb(II) ions had already been removed from the solution and that further addition of adsorbent did not significantly enhance the process. Therefore, 50 mg was selected as the optimum adsorbent mass, providing the highest Pb(II) removal efficiency of 99.5 ± 2.7% while avoiding unnecessary excess use of the nanocomposite.

The high removal efficiency obtained at 50 mg can be linked to the multifunctional structure of the MnO_2_/NH_2_-MIL-101(Fe)/*g*-C_3_N_4_ nanocomposite. MnO_2_ offers abundant hydroxyl- and oxygen-containing surface groups that can interact with Pb(II) ions through surface complexation and ion-exchange pathways. Meanwhile, NH_2_-MIL-101(Fe) contributes a porous framework containing amine groups and Fe–O clusters, which can provide additional coordination sites and facilitate the diffusion of Pb(II) into the internal pores. The g-C_3_N_4_ component further enriches the composite surface with nitrogen-containing moieties, such as amino and pyridinic-like nitrogen sites, which can act as electron-donating centers for Pb(II) chelation. The combination of these active functionalities creates an integrated adsorption environment involving electrostatic attraction, coordination bonding, surface complexation, and pore-assisted uptake. At 50 mg, these binding sites are present in sufficient quantity to achieve nearly complete Pb(II) removal, whereas higher dosages provide redundant adsorption sites with little additional benefit.

#### 3.2.4. Impact of the Background Ions

The influence of background electrolytes on Pb(II) adsorption by the MnO_2_/NH_2_-MIL-101(Fe)/g-C_3_N_4_ nanocomposite was examined at pH 6 using NaCl, Na_2_SO_4_, and NaH_2_PO_4_ as representative coexisting salts. Real wastewater usually contains different inorganic ions that may interfere with heavy metal adsorption by competing for active sites, changing the ionic strength of the solution, or modifying the aqueous speciation of metal ions. As shown in Figure 6b, Pb(II) removal remained high in all tested systems, confirming that the nanocomposite retained strong adsorption ability even in the presence of background ions. In the absence of added electrolyte, the removal efficiency was about 99.5%. After electrolyte addition, a slight decrease was observed, particularly at higher ionic concentrations. This decrease can be attributed mainly to electrostatic shielding and partial competition between coexisting ions and Pb(II) for accessible binding sites on the nanocomposite surface. Nevertheless, the removal efficiency remained above 95% even at 0.2 mmol L^−1^ electrolyte concentration, indicating good tolerance of the adsorbent toward common dissolved ions.

The extent of inhibition depended on both the type and concentration of the background electrolyte. Increasing the electrolyte concentration from 0.1 to 0.2 mmol L^−1^ generally reduced Pb(II) removal, indicating that higher ionic strength weakens the interaction between Pb(II) species and the active surface sites of the nanocomposite. Among the tested salts, Na_2_SO_4_ caused the most noticeable decrease, with Pb(II) removal declining to approximately 95.5% at 0.2 mmol L^−1^. This stronger effect may be related to the divalent nature of SO_4_^2−^, which can interact more strongly with Pb(II) in solution and may also compete with Pb(II)-binding sites, especially those associated with Fe–O clusters and Mn–OH/Mn–O groups [70,71]. NaCl showed a moderate influence, where Pb(II) removal decreased slightly as Cl^−^ concentration increased, likely because chloride ions can partially screen surface charge and form weak aqueous Pb–Cl species. In contrast, NaH_2_PO_4_ exhibited the weakest inhibitory effect, and the removal efficiency remained close to 98–99% even at the higher concentration. This may be due to the lower effective charge of H_2_PO_4_^−^ compared with SO_4_^2−^ and the possible contribution of phosphate-related Pb(II) complexation or surface-assisted immobilization [72]. Overall, the inhibitory effect followed the order Na_2_SO_4_ > NaCl > NaH_2_PO_4_, suggesting that Pb(II) adsorption by MnO_2_/NH_2_-MIL-101(Fe)/g-C_3_N_4_ is more sensitive to strongly coordinating or divalent anions, while the composite still maintains excellent removal performance in complex ionic media.

### 3.3. Selectivity Dynamics and Competitive Binding Affinity

**Figure 7:**
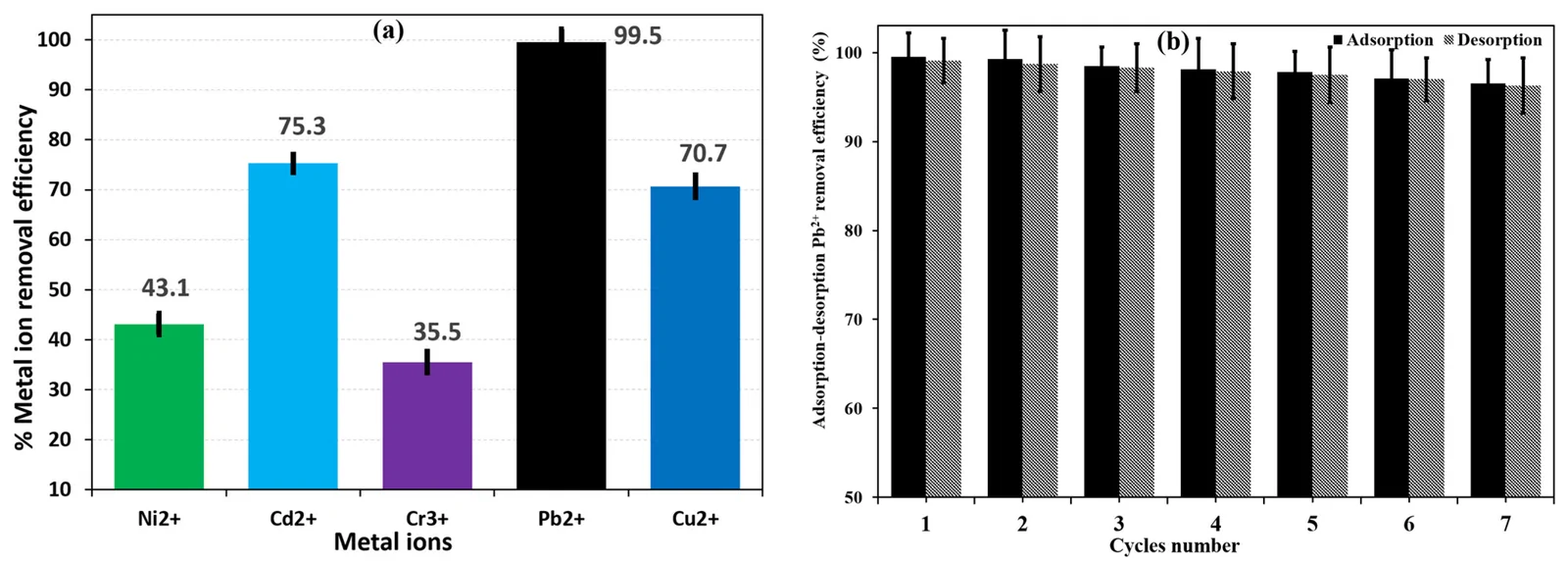
Selectivity towards Pb^2+^ in the presence of other metal ions (**a**) and regeneration capacity (**b**) of the MnO_2_/NH_2_-MIL-101(Fe)/g-C_3_N_4_ at the optimum conditions.

Evaluating the competitive binding profiles of the ternary MnO_2_/NH_2_-MIL-101(Fe)/*g*-C_3_N_4_ framework reveals a clear preference for target Pb^2+^ ions when evaluated in multi-element matrices containing co-existing competing cations (Cu^2+^, Cd^2+^, Ni^2+^, and Cr^3+^) at an operational pH of 6.0 (Figure 7a). Under these competitive conditions, the nanocomposite maintains a highly targeted lead removal efficiency of 99.5 ± 2.7%.

This pronounced selectivity is governed directly by the matching hard/soft coordination fields and the geometric configurations across the hybridized network:

(i) *Hard/Soft Lewis acid-base affinity*: Pb^2+^ behaves as a highly polarizable, soft Lewis acid. This configuration matches perfectly with the soft electron-donating sites distributed across the hybrid interface, specifically the lone pairs on the aromatic triazine nitrogen rings (-C=N-) of the *g*-C_3_N_4_ sheets, the exocyclic primary amine configurations (-NH_2_) decorating the MOF cages, and the polarizable surface hydroxyl species (Mn-OH) on the manganese dioxide fractions.
(ii) *Dehydration and pore accessibility*: The relatively large ionic radius (1.19 °A) and low hydration energy of Pb^2+^ allow it to shed its surrounding water shell far more easily than smaller, highly hydrated transition metals. This allows the bare lead ions to diffuse rapidly into the unblocked, three-dimensional porous channels of the iron framework without encountering early energy barriers.
(iii) *Competitive cation profiles*: In comparison, co-existing soft transition ions like Cd^2+^ and Cu^2+^ achieved respectable extraction metrics of 75.3% and 70.7%, respectively, reflecting the material’s secondary broad-spectrum affinity for transition metals. Conversely, the smaller, harder, and highly hydrated ions, specifically Cr^3+^ (35.5%) and Ni^2+^ (43.1%), exhibited a severe drop in uptake. This weak performance stems from the high energy required to strip their tightly bound hydration shells, alongside unfavorable binding enthalpies and steric exclusion within the narrower pore gaps.

Critically, the high selectivity of the material is closely linked to its structural stability. The integrated three-dimensional NH_2_-MIL-101(Fe) scaffolding acts as a robust mechanical core that permanently holds the active *g*-C_3_N_4_ sheets and nanoscale MnO_2_ fractions in a highly dispersed layout. By preventing these phases from stacking or aggregating, the structural configuration ensures that all coordination sites remain open and active, while protecting the core metal–ligand coordination sites from chemical degradation during competitive extraction. These findings prove that combining N-donor binding sites with stable iron/manganese oxides creates a robust inner-sphere chelation environment, establishing this ternary hybrid as a highly efficient and stable choice for selective Pb^2+^ ion removal.

### 3.4. Recycling and Regeneration Capacity of the Adsorbent MnO_2_/NH_2_-MIL-101(Fe)/g-C_3_N_4_

The long-term economic viability and structural durability of an engineered nanomaterial are determined by its capacity for repetitive regeneration without suffering significant drops in removal efficiency. To evaluate these properties, cyclic adsorption–desorption runs were conducted on the ternary MnO_2_/NH_2_-MIL-101(Fe)/g-C_3_N_4_ (Figure 7b). Following initial Pb^2+^ capture, the loaded composite was collected via centrifugation, washed thoroughly with deionized water to eliminate non-specifically bound surface fractions, and subsequently treated with a mixed stripping solution of 0.1 *M*-HCl and 0.05 *M*-EDTA under mild ultrasonication for 5.0 min. The pairing of hydronium ions (H_3_O^+^) and EDTA molecules drive rapid desorption: the high concentration of protons protonates the framework’s exocyclic amine (-NH_2_) and triazine (C=N-) groups, causing strong electrostatic repulsion that breaks the dative bonds with Pb^2+^, while the free EDTA acts as a strong competing ligand that encapsulates the detached lead ions. The regenerated solid was rinsed back to a neutral pH and dried at 60 °C before being reintroduced into subsequent batch experiments. Figure 7b presents the adsorption efficiency (%) of the ternary nanocomposite across seven consecutive recycling cycles. The ternary hybrid material retains an outstanding performance of 91.3% after seven complete recycling cycles, proving the excellent structural reusability and high stability during repetitive chemical treatments. Post-cycling evaluation via FTIR analysis verified that the primary vibrational peaks for the carboxylate groups, framework amine functions, and triazine rings match the pristine material’s baseline coordinates perfectly. This high chemical stability is a direct consequence of the intentional multi-component design:

(i) The three-dimensional NH_2_-MIL-101(Fe) framework forms a robust structural skeleton that prevents the mechanical collapse or shedding of the active phases during repeated washing and sonicating steps.
(ii) The underlying MOF channels are structurally reinforced by the integrated *g*-C_3_N_4_ polymer layers, creating a protective interface that minimizes the localized dissolution of iron nodes in mildly acidic stripping media.
(iii) This spatial isolation keeps the MnO_2_ NPs highly dispersed, avoiding the typical loss of active surface area caused by particle aggregation.

By preventing structural collapse and shielding the active coordination sites, this unique interfacial arrangement ensures high mass-transport rates and stable binding affinities across multiple cycles. These findings confirm that the ternary nanocomposite is a robust, highly stable, and commercially viable candidate for continuous heavy metal remediation from wastewater streams.

Despite the promising Pb(II) adsorption performance of the MnO_2_/NH_2_-MIL-101(Fe)/g-C_3_N_4_ nanocomposite, several limitations should be considered when assessing its practical applicability. First, the adsorption experiments were conducted mainly under batch conditions, and therefore the behavior of the material under continuous-flow or fixed-bed column operation has not yet been established. Such studies will be important for evaluating mass-transfer behavior and operational performance under more realistic treatment conditions. Second, although the nanocomposite showed satisfactory regeneration over the investigated cycles, its long-term structural stability and adsorption efficiency over a larger number of adsorption–desorption cycles remain to be verified.

### 3.5. Kinetic Study and Effect of Time

**Figure 8:**
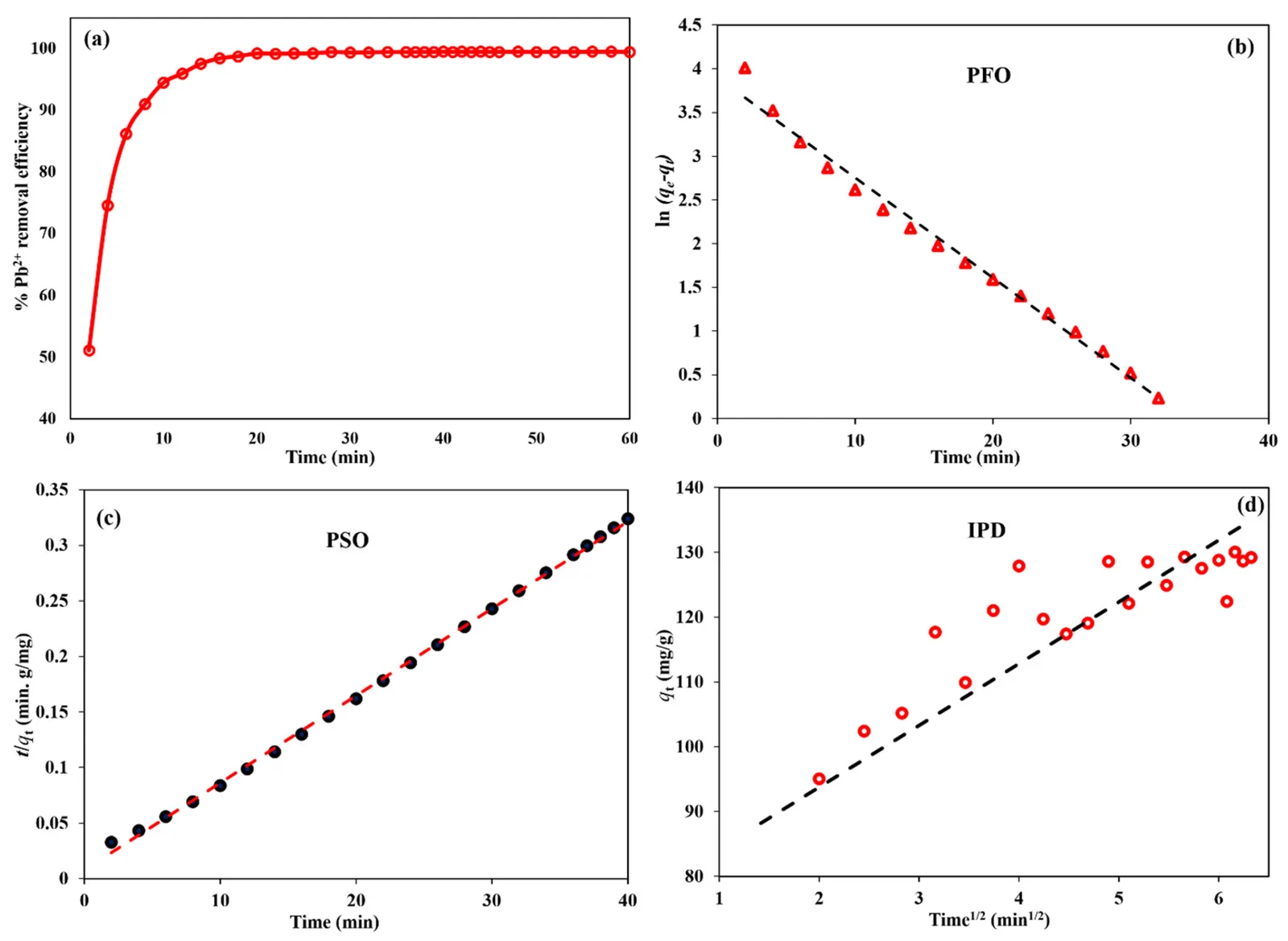
Impact of contact time (**a**), PFO (**b**), PSO (**c**) and IPD (**d**) models for the Pb^2+^ adsorptive removal by MnO_2_/NH_2_-MIL-101(Fe)/*g*-C_3_N_4_ nanocomposite.

The influence of contact time on Pb(II) adsorption by the MnO_2_/NH_2_-MIL-101(Fe)/g-C_3_N_4_ nanocomposite was investigated at pH 6 using 50 mg of adsorbent, and the obtained kinetic profiles are shown in Figure 8. As illustrated in Figure 8a, Pb(II) removal proceeded very rapidly during the early stage of adsorption, increasing sharply within the first few minutes and exceeding 90% after approximately 8 min. This fast uptake can be attributed to the large number of readily accessible active sites on the nanocomposite surface, including oxygen-containing groups of MnO_2_, amino groups of NH_2_-MIL-101(Fe), and nitrogen-rich sites of g-C_3_N_4_, which can interact efficiently with Pb(II) ions through electrostatic attraction, surface complexation, and coordination/chelation. After the initial rapid stage, the adsorption rate gradually decreased as the available binding sites became occupied, and the system approached equilibrium at around 40 min, with an experimental adsorption capacity of approximately 124 mg.g^−1^. To further clarify the adsorption behavior, the kinetic data were fitted using the linear pseudo-first-order (PFO), pseudo-second-order (PSO), and intraparticle diffusion (IPD) models [73,74,75]. The PFO model, which assumes that the adsorption rate depends mainly on the number of unoccupied surface sites, gave a moderate fit with *q*_e_ = 132 mg.g^−1^, k_1_ = 0.0045 min^−1^, *R*^2^ = 0.9562, and RMSE = 2.914 mg.g^−1^ (Table 1, Figure 8b). In contrast, the PSO model (Figure 8c), which considers the adsorption rate to be controlled by interactions between Pb(II) ions and active surface sites, provided the best description of the experimental data, as indicated by the highest *R*^2^ value of 0.9938 and the lowest RMSE of 1.100 mg.g^−1^. Moreover, the calculated *q*_e_ value from the PSO model, 123.48 mg.g^−1^, was very close to the experimental equilibrium capacity, confirming the suitability of this model for describing Pb(II) uptake. These results suggest that chemisorption-related interactions, including complexation with oxygenated groups, coordination with amino sites, and electrostatic attraction, play a major role in the adsorption process. The IPD model was also applied to assess the contribution of pore diffusion. The relatively low *R*^2^ value of 0.5931 and high RMSE of 8.882 mg.g^−1^ indicate that intraparticle diffusion alone cannot adequately describe the adsorption kinetics (Table 1, Figure 8d). In addition, the large intercept value, *C* = 84.161 mg.g^−1^, reflects a significant boundary-layer effect, while the diffusion constant *K*_id_ = 7.443 mg.g^−1^ min^−0.5^ suggests that pore diffusion contributes only partially after the rapid surface adsorption stage. Overall, the kinetic analysis confirms that Pb(II) adsorption onto the MnO_2_/NH_2_-MIL-101(Fe)/*g*-C_3_N_4_ nanocomposite is a fast and efficient process dominated mainly by surface-controlled chemisorption, with a secondary contribution from diffusion into the porous structure of the nanocomposite.

### 3.6. Equilibrium Adsorption Isotherms

To understand the surface energetic distribution and pathways governing Pb^2+^ isolation, the equilibrium data collected across a concentration gradient (5–100 mg L^−1^) were modeled using non-linear Langmuir and Freundlich isotherm frameworks [76,77]. Statistical metrics reveal that the non-linear Langmuir model provides a more accurate fit for the experimental data than the Freundlich relationship. This preference is demonstrated by its superior correlation coefficients (*R*^2^ > 0.9985) and minimized residual fitting errors. This mathematical preference suggests that the immobilization process is governed by monolayer chemisorption happening on a fixed number of identical, localized surface sites, without any significant multilayer contribution. The non-linear regression parameters derived from both models are organized in Table 2. The high maximum monolayer capture capacity (*q*_max_) was determined to be 431.8 mg.g^−1^ under optimal operating conditions (pH 6.0 at 25.0 °C). This high capacity highlights the clear structural advantages of the multi-component architecture. Additionally, the calculated dimensionless separation factor (*R*_L_) is 0.2631. Because this value falls within the classic 0 < *R*_L_ < 1 boundary, it confirms that the extraction process is thermodynamically highly favorable. In comparison, the Freundlich model yields a lower correlation coefficient (*R*^2^ = 0.9068), showing that macroscopic surface heterogeneity plays a secondary role in the process. However, the calculated heterogeneity factor (*n* = 4.9062) sits well above unity, confirming that adsorption remains highly favorable even across localized energy gradients.

The structural characteristics of each component directly explain these exceptional equilibrium metrics: firstly, the iron-based NH_2_-MIL-101(Fe) framework provides an open, three-dimensional mesoporous network that avoids pore blockages and maximizes fluid mass transfer. This structural skeleton distributes the active sites uniformly, ensuring high spatial accessibility. Secondly, the extensive monolayer capacity is further enhanced by the integrated MnO_2_ nanoparticles and *g*-C_3_N_4_ layers, which add a high density of specialized binding sites. These include active surface hydroxyl positions (Mn-OH), electron-rich primary amine groups (-NH_2_), and aromatic triazine nitrogen rings (-C=N). Because the underlying MOF scaffolding keeps these components highly dispersed, it prevents the natural self-aggregation of MnO_2_ and stops the layer-stacking of *g*-C_3_N_4_. This ensures that the dense collection of active sites remains fully exposed to the liquid phase. Ultimately, the high capacity of 431.8 mg.g^−1^ is driven by a coordinated combination of open pore diffusion, inner-sphere surface complexation, exocyclic amine chelation, and oxygen-vacancy trapping along a highly accessible monolayer interface.

### 3.7. Proposed Adsorption Mechanisms

The high Pb^2+^ immobilization capability displayed by the ternary MnO_2_/NH_2_-MIL-101(Fe)/*g*-C_3_N_4_ nanocomposite is driven by a cooperative, multi-modal network of interfacial capture mechanisms. Rather than operating as isolated components, the deliberate hybrid design establishes structural interactions where each specific phase creates highly accessible, target-specific extraction pathways.

The individual mechanical roles and structural contributions of each constituent phase are defined as follows:

(i) *The MnO_2_ NPs phase*: This component acts as a highly active chemical trapping zone, relying heavily on its structural density of surface defect sites and oxygen vacancies. At optimized aquatic parameters (pH 5.0–6.0), its abundant, reactive surface hydroxyl groups (Mn-OH) undergo localized ion-exchange and inner-sphere surface complexation to capture heavy metal ions. Concurrently, the intrinsic layer gaps within the oxide matrices facilitate partial interlayer ion accommodation and localized chemical trapping within the oxygen vacancies.
(ii) *The NH_2_-MIL-101(Fe) MOF*: Operating as the foundational structural skeleton of the composite, this component provides an ultrahigh internal specific surface area and an open, three-dimensional mesoporous network. This open channel configuration accelerates fluid mass transfer, rapidly guiding hydrated lead ions directly into the core of the hybrid material. Once inside, the heavy metals are immobilized via strong coordination bonds with the electron-rich exocyclic amine groups (-NH_2_) and dative interactions with the exposed open iron-oxygen (Fe-O) cluster nodes.
(iii) *The g-C_3_N_4_ polymer sheets*: This carbon-nitride phase provides a dense network of conjugated, electron-donating nitrogen sites. Divalent lead ions form stable coordination complexes by interacting with the available lone pairs on the aromatic pyridinic nitrogen centers (-C=N). Simultaneously, the highly delocalized π-electron clouds across the heptazine rings alter the localized surface charge density, establishing long-range cation-π attractions and structural stabilization that firmly anchor the bound metal complexes.

Ultimately, these individual chemical pathways work in unison to maximize the overall decontamination efficiency. The highly porous 3D MOF architecture prevents the natural self-aggregation of the MnO_2_ fractions and stops the dense layer-stacking of the 2D g-C_3_N_4_ sheets. By holding these structures apart, the composite architecture keeps the entire density of active binding sites exposed to the liquid phase. The resulting remediation mechanism represents a highly coordinated combination of open pore diffusion, electrostatic attraction, inner-sphere surface complexation, exocyclic amine chelation, and oxygen-vacancy trapping. This cooperative behavior explains the outstanding Pb^2+^ removal metrics, establishing this ternary framework as an advanced reference standard for multi-functional wastewater cleanup.

## 4. Conclusions

In this work, a ternary MnO_2_/NH_2_-MIL-101(Fe)/*g*-C_3_N_4_ nanocomposite was successfully synthesized and applied as an efficient adsorbent for Pb^2+^ removal from aqueous media. The formation of the hybrid structure was verified using FTIR, PXRD, Raman spectroscopy, XPS, BET analysis, TEM, SEM, and EDX, confirming the integration of MnO_2_, amine-functionalized MIL-101(Fe), and graphitic carbon nitride within a hierarchical porous architecture. This structural arrangement combines the accessible mesoporous channels of NH_2_-MIL-101(Fe), the oxygen-rich surface of MnO_2_, and the nitrogen-containing domains of *g*-C_3_N_4_, thereby providing a high density of exposed binding sites for Pb^2+^ uptake. Under optimized conditions of pH 6.0, 50 mg adsorbent dosage, and 40 min contact time, the nanocomposite achieved a Pb^2+^ removal efficiency of 99.5 ± 2.7% and a maximum Langmuir adsorption capacity of 431.8 mg.g^−1^. Kinetic analysis showed that the adsorption process was better described by the pseudo-second-order model, suggesting that surface-controlled interactions play a dominant role, while intraparticle diffusion contributes as an additional transport step. The equilibrium data followed the Langmuir model more closely than the Freundlich model, indicating a dominant monolayer adsorption behavior over highly accessible active sites.

The high Pb^2+^ affinity of the MnO_2_/NH_2_-MIL-101(Fe)/*g*-C_3_N_4_ nanocomposite can be attributed to the cooperative contribution of multiple surface functionalities, including Mn–OH groups, Fe–O clusters, carboxylate groups, exocyclic –NH_2_ groups, Fe–N linkages, and the C–N/C=N units of *g*-C_3_N_4_. These sites promote Pb^2+^ capture through electrostatic attraction, surface complexation, coordination/chelation, oxygen-vacancy trapping, and pore-assisted diffusion. Competitive adsorption experiments further demonstrated the preferential uptake of Pb^2+^ over Cr^3+^, Ni^2+^, Cu^2+^, and Cd^2+^, highlighting the selectivity of the hybrid surface toward lead ions. The nanocomposite also maintained high adsorption performance in the presence of common background electrolytes, although sulfate ions produced the most noticeable inhibitory effect. In addition, regeneration experiments showed that the adsorbent retained 91.3% removal efficiency after seven adsorption–desorption cycles, indicating good reusability and structural stability. Overall, the results demonstrate that the deliberate integration of MnO_2_, NH_2_-MIL-101(Fe), and *g*-C_3_N_4_ offers an effective strategy for designing multifunctional MOF-based adsorbents with strong Pb^2+^ affinity, high adsorption capacity, and practical potential for heavy metal remediation in aqueous systems.

## Figures and Tables

**Table 1:** Linear kinetic equations for the sorption of Pb^2+^ ions on MnO_2_/NH_2_-MIL-101(Fe)/g-C_3_N_4_ nanocomposite at 25.0 °C, for 25 mgL^−1^ as initial metal ion concentration, pH 6.0 and 50 mg nanocomposite mass.

Kinetic Model	Sorption Linear Kinetic Equations	Calculated Parameters	
Pseudo-first order (PFO)	$\ln\left(q_{e\;}-q_{t}\right)={lnq}_{e}-k_{1}\;t$	*q*_e_ (mg.g^−1^)	132

		*k*_1_ (min^−1^)	0.0045
		*R* ^2^	0.956196
		*RMSE* (mg.g^−1^)	2.914
Pseudo-second order (PSO)	$\frac{t}{q_{t}}=\;\frac{1}{k_{2}q_{e}^{2}\;t}+\frac{t}{q_{e}}\;$		123.48
		*q*_e_ (mg.g^−1^)	

		*k*_2_ (g mg^−1^ min^−1^)	0.343
		*R* ^2^	0.99376
		*RMSE* (mg.g^−1^)	1.100
Intraparticle Diffusion (IPD)	$q_{t}=K_{id}\;t^{0.5}+C$	*K*_id_ (mg/(g·min^0.5^)	7.443
		*C* (mg.g^−1^)	84.161
		*R* ^2^	0.6931
		*RMSE* (mg.g^−1^)	8.882

*t* = time (minutes), *q*_t_ = the milligrams of Pb^2+^ adsorbed per one gram of the developed nanocomposite at time *t* (mg/g). *C* = a constant that reflects the thickness of the boundary layer; *K*_id_ = the intraparticle diffusion rate constant (mg/g min^0.5^).

**Table 2:** Non-linear Langmuir and Freundlich isotherm parameters modeled for the equilibrium adsorption of Pb^2+^ ions onto MnO_2_/NH_2_-MIL-101(Fe)/*g*-C_3_N_4_ nanocomposite (initial concentration of Pb^2+^ 5–100 mg.L^−1^, 50 mg nanocomposite mass, pH 6.0 at 25.0 °C).

Isotherm Model and Parameters	
Langmuir model	$q_{e}=\frac{q_{m}K_{L}C_{e}}{1\;+\;K_{L}C_{e}}$
*q*_m_ (mg.g^−1^)	431.8
*K*_L_ (L.mg^−1^)	0.150
*R* ^2^	0.9985
*R_L_*	0.2631
*RMSE*	4.151
Freundlich model	qe=KF Ce1n
*K*_F_ (mg^1−n^·L^n^·g^−1^)	167.5
*n*	4.9062
*R* ^2^	0.9068
*RMSE*	14.71

## Data Availability

The original contributions presented in this study are included in the article. Further inquiries can be directed to the corresponding author.
